# Structural equation modeling for identification of patient safety antecedents in primary care

**DOI:** 10.1186/s12875-021-01533-6

**Published:** 2021-09-13

**Authors:** Katarzyna Kosiek, Iwona Staniec, Maciej Godycki-Cwirko, Adam Depta, Anna Kowalczyk

**Affiliations:** 1 Family Doctors’ Clinic, Pomorska 96, 91-402 Lodz, Poland; 2 Department of Management, Lodz University of Technology, Lodz, Poland; 3 Centre for Family and Community Medicine, Faculty of Health Sciences, Medical University of Lodz, Kopcinskiego 20, 90-153 Lodz, Poland; 4 Department of Medical Insurance and Health Care Financing, Medical University of Lodz, Lodz, Poland

**Keywords:** Primary care, Patient safety, SEM, Antecedents of patient safety

## Abstract

**Background:**

Patient safety is defined as an activity that minimizes and removes possible errors and injuries to patients. A number of factors have been found to influence patient safety management, including the facilities available in the practice, communication and collaboration, education regarding patient safety and generic conditions. This study tested a theoretical model of patient safety interventions based on safety antecedents.

**Methods:**

Medical professionals were surveyed using a questionnaire developed by Gaal et al. The results were analyzed with SPSS 20 and AMOS. A hypothetical model of direct and indirect effects on patient safety in a primary care environment was created and analyzed using structural equation modeling (SEM).

**Results:**

SEM proved to be an effective tool to analyse safety in primary care. The facilities in the practice appear to have no significant influence on patient safety management in the case of female respondents, those below mean age, those who are not GPs (general practitioner) and respondents not working in counselling centres.

**Conclusions:**

The integrated safety model described in the study can improve patient safety management.

**Supplementary Information:**

The online version contains supplementary material available at 10.1186/s12875-021-01533-6.

## Background

In this study, patient safety is understood as an activity that minimizes and removes possible errors and injuries to patients. In this sense, it comprises prevention of medical errors and avoidable adverse events, the protection of patients from harm or injury, collaborative efforts by individual healthcare providers and a strong, well‐integrated healthcare system [1]. However, in the US alone, approximately 5.08% of all adult outpatients, i.e. 12 million adults, experience diagnostic errors every year. It has been estimated that about half of these errors could potentially be harmful [2].

In Poland, patient safety research is scarce. Only general data has been collected. In one region of Poland, 5748 out of 8062 patients’ complaints regarding medical care cases in the years 2006–2008 were recognized as medical errors [3]. To avoid errors and improve patient safety, health care organizations need to implement corrective activity. Despite increasing understanding of patient safety strategies, little is known about the precedents for improving safety in primary care.

A number factors have been found to influence patient safety management strategies, including the facilities available in the practice, communication and collaboration, education regarding patient safety and generic conditions [4, 5]. The present study examines these factors, and some others, with the aim of developing more effective interventions and identifying routes to implementing better strategies.

The principle of concept analysis states that identifying the antecedents and consequences would help in that [6, 7]. Certain antecedents have already been found to positively influence patient safety in primary care [8–10]: documentation of safety incidents, provision of adequate staffing and facilities in the practice, encouraging communication and collaboration to coordinate safe patient care, provision of in-service education and training for providers and fostering a safety culture among leaders and providers. For the purposes of the present study, these antecedents were classified into four strategies: generic conditions (GC), facilities in the practice (FP), communication and collaboration (CC) and education regarding patient safety (EPS).

Patient safety is needed at all levels of the healthcare system. Regardless of the national setting, a positive safety culture focuses on collective improvement and teamwork [11, 12]. Technology and expertise exist to manage these conditions and limit their impact on patient safety [7, 13]. However, safety problems are most effectively managed through education, appropriate facilities and communication.

## Objective

The aim of the present study was to test a theoretical model of patient safety interventions based on safety antecedents.

## Methods

**Fig. 1:**
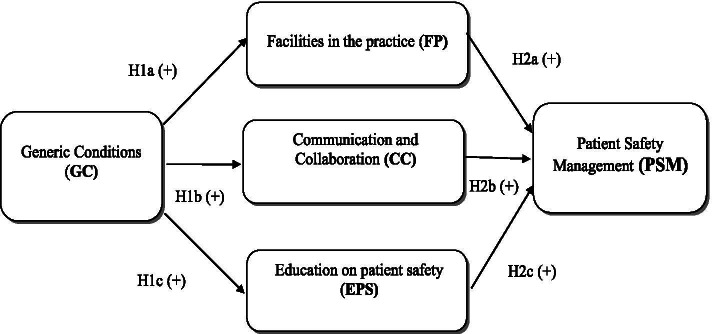
Research conceptual model

The patient safety strategies included in the present study were adopted from earlier Dutch research [1], examining the variables affecting patient safety management (PSM): facilities in the practice, communication and collaboration, education regarding patient safety. Therefore, it is assumed that (Fig. 1):

- *H1: Common perception generic conditions for patient safety are antecedents of: (a) facilities in the practice, (b) communication and collaboration and (c) education on patient safety).*
- *H2: The three strategies (a) facilities in the practice, (b) communication and collaboration and (c) education on patient safety are antecedents of patient safety management.*

The population used in this study was varied (Table 1). We check whether the control variables have no influence on the measured effect (Table A2, Supplementary Information) [14], it is assumed that:

- *H3: The relationships with strategies in all subgroups studied are identical.*

**Table 1:** Sample profile (*N* = 248)

Characteristics	n	%
Sex		
Male	132	53.2
Female	116	46.8
Age [value from 29 to 63], mean and standard deviation	43	7
Current professional discipline		
GP (general practitioner)	141	56.9
Internist	102	41.1
Other primary care physician	51	20.6
Medical teacher	10	4
Scientific researcher	10	4
Other	6	2.4
Current professional discipline^a^		
Individual	61	24.6
Group	53	21.4
Counseling centers	164	66.1
Number of patients per respondent [value from 0 to 4,000], mean (standard deviation)	1,763	872.96
Number of patients in the facility [value from 300 to 16,220], mean and standard deviation	5,091	3,095.5
Area of practice		
City with over 100,000 inhabitants	123	49.6
City of 30,000 to 100,000 inhabitants	37	14.9
City with less than 30,000 inhabitants	44	17.7
Small town / village	44	17.7

^a^Some respondents were specialists in more than one discipline

The model was analyzed using structural equation modeling (SEM): an extension of linear regression that enables the modeling of complex relationships between interrelated (and thus correlated) variables. The resulting models account for direct and indirect impacts and can cope with correlated variables. It can also be used to model causal relationships. In SEM, it is possible to use more than one attribute to characterize latent variables. SEM uses an optimization process to create an index score for each latent variable using linear regression.

In this study, SEM was used to characterize latent variables and to produce a causal model incorporating several of them. The models allow the diagnosis and assessment of potential causes of safety management failure, as well as the identification of relevant management methods.

### Participants

Participants were recruited from several conferences and seminars in Poland where the strategies were presented; most of respondents were physicians potentially interested in patient safety. Further contacts were recruited through a snowball sampling procedure. In order to obtain the reliability of the answers, context descriptions were provided. Participation in the study was voluntary. A professional research and consulting company collected the questionnaires from the Polish respondents. During March 2019, 251 replies were received from1300 targeted respondents. Out of these 251 replies, three were not completed and were not included in the analysis. Therefore, the questionnaire was completed by 248 individuals (Table 1).

The obtained data was monitored manually. If the questionnaire was not completed correctly, or not at all, the respondent was contacted. We confirmed our understanding of the individual responses.

The respondents differed significantly with regard to the number of patients cared for one physician (ranging from 0 to 4,000; mean 1.763 patients; SD = 873) and the size of population of their counseling centers (ranging from 300 to 16,220 patients; mean 5091; SD = 3.096). In addition, 49.6% of respondents conducted their medical practice in a city with over 100,000 people.

The validity of the questionnaire was not evaluated because it had been adapted from a previous study [5]. The questionnaire was designed to identify factors influencing patient safety in primary care. It comprised 38 items which were adopted from multiple scales used elsewhere in the literature. The instrument comprises the following five constructs that were analyzed by SEM: patient safety management, facilities in the practice, generic conditions, communication and collaboration, and education. A pilot test with 10 participants was run to remove irrelevant or weak questions. The collected data was carefully cleaned before analysis. Principal Component Analysis (PCA) was used to identify the primary components that were measured with the survey questions. Internal consistency was checked with a standard Cronbach’s Alpha test.

### Measurement of latent variables

The instrument used in the study consists of 38 items measuring the five latent variables (strategies). All measures are scored on a four-point Likert scale providing sufficient variance and covariance for better data analysis [15]. In addition, all the manifest variables included in the instrument, i.e. the items, reflect the changes of their corresponding latent variables and therefore can be seen as being caused by constructs [16]. In addition, all the constructs are operationalized as first-order latent variables to reduce the complexity of the whole model, as the number of latent variables did not increase. In addition, as no blocks of indicators were found to share specific common characteristics, all items were treated as a single latent variable. A more detailed specification of items is presented in Supplementary Information Table A1.

Unrotated principal component factor analysis (CFA), principal component analysis with varimax rotation, and principal axis analysis with varimax rotation all revealed the presence of three distinct factors with eigenvalue greater than 1.0, rather than a single factor [17]. The seven factors together accounted for 67.84 percent of the total variance; the first (largest) factor did not account for a majority of the variance (11.324%). While the results of these analyses do not preclude the possibility of common method variance, they do suggest that common method variance is not of great concern and thus is unlikely to confound the interpretations of results.

After 248 observations, the accuracy of each of the hidden variables was found to be at least 0.7, as measured by Cronbach’s alpha: each variable also demonstrated composite reliability (CR) of > 0.7 and average variance extracted (AVE) of > 0.5. The latent variable was not constructed from all of the observable variables proposed by Gaal, Verstappen and Wensing [5]. The strength of these considerations deliberations is to meet the threshold conditions by the constructed variables and the observable variables representing them.

The square root of the AVEs are compared with the appropriate correlation factors in Table 2. They have much higher values, indicating positive divergent validity, i.e. the individual latent variables differ significantly from one another. In addition, discriminant validity analysis was performed to determine whether the measures of each construct differ sufficiently from those of other constructs [18].

**Table 2:** Discriminant validity for constructs and their correlations

	Mean	Standard deviation	*R*^2^	FP	CC	PSM	GC	EPS
FP	1.493	.591	.356	.711				
CC	1.221	.522	.412	.488	.927			
PSM	1.529	.653	.365	.543	.688	.710		
GC	1.245	.466	.356	.503	.590	.555	.709	
EPS	1.077	.365	.593	.408	.486	.615	.627	.710

*CC* Communication and collaboration, *EPS* Education on patient safety, *FP* Facilities in the practice, *GC* Generic conditions, *PSM* Patient safety management, *R*^*2*^ Coefficient of determination, the square root value of AVE is shown on the diagonal, under the diagonal of the Pearson correlation coefficient. For all *p* < 0.001

The part of the model that examines relationship between the latent variables and their measures is known as the measurement model. A previous CFA based on a sample of respondents in Poland confirmed that the measurement model demonstrates satisfactory construct validity, discriminant validity and internal consistency [19, 20]. In the case of the measurement models, there is no reason to reject the hypothesis that the standardized residual values of the empirical and theoretical matrix are equal to zero (χ2 = 564.812; *p* = 0.000). The model was found to demonstrate a good fit to the data, as indicated by a root mean square of approximation error (RMSEA) of 0.061 < 0.08 (LO = 0.054; HI = 0.069), and to demonstrate good acceptability, as indicated by χ2/ss = 1.928 < 2, GFI = 0.951 > 0.9 and AGFI = 0.921 > 0.9 [19, 21, 22]. All latent variables in the model are significantly correlated, as shown in Table 3.

**Table 3:** Covariance and correlation between latent variables

Relation			Covariances	S.E.	C.R.	Correlations
EPS	< – >	CC	.127	.029	4.428	.486***
EPS	< – >	PSM	.119	.023	5.302	.615***
EPS	< – >	FP	.138	.031	4.491	.408***
CC	< – >	PSM	.125	.027	4.595	.688***
CC	< – >	FP	.154	.036	4.234	.488***
PSM	< – >	FP	.127	.027	4.790	.543***
EPS	< – >	GC	.189	.040	4.790	.627***
CC	< – >	GC	.167	.041	4.019	.590***
PSM	< – >	GC	.116	.028	4.161	.555***
FP	< – >	GC	.183	.045	4.109	.503***

*CC* Communication and collaboration, *CR* Composite reliability, *EPS* Education on patient safety, *FP* Facilities in the practice, *GC* Generic conditions, *PSM* Patient safety management

^***^ mean *p* < 0.0001

## Results

SEM enabled patient safety management to be modelled using causal models derived by experts and validated using monitoring survey data. Once defined, SEM was used to regress the defined strategies against each other to produce final model outputs. As a result, a series of causal relationship arrows are produced; each was associated with a standard coefficient representing its impact on a scale of -1 (high negative impact) to + 1 (high positive impact) [18, 21]. This model was estimated using the maximum likelihood method, assuming a multidimensional normal distribution. No suspicious response pattern was observed, and, following the outlier labeling rule, no significant outlier was observed; however, as no skewness or kurtosis values higher than one were found, the data was normal. As the sample size was small, analysis was based on the residual bootstrap method and the sandwich standard error estimator. In the case of the structural models, there is no reason to reject the hypothesis that the standardized residual values of the empirical and theoretical matrix are equal to zero (χ2 = 582.298; *p* = 0.000). The value of root mean square of approximation error (RMSEA) is 0.063 < 0.08 (LO = 0.055; HI = 0.070), standardized RMR = 0.0762 < 0.08 indicates a good fit of the model. The values of χ2/ss = 1.981 < 2 indicate the acceptability of the model. Indices GFI = 0.947 > 0.9 and AGFI = 0.917 > 0.9 assume the values are acceptable, so the model is well suited to the data and can be used in the description [22]. Following the criteria and rules of thumb specified by [23], data analysis results are interpreted in the following sections in terms of model estimations.

The determine the predictive accuracy of the model, the determination coefficients (*R*^2^) of the latent variables were estimated. *R*^2^ values higher than 0.33 are considered acceptable for latent variables [24, 25]. The *R*^2^ statistics were found to be significantly higher than 0.33 (Table 3) confirming that the model has sufficient predictive accuracy.

**Fig. 2:**
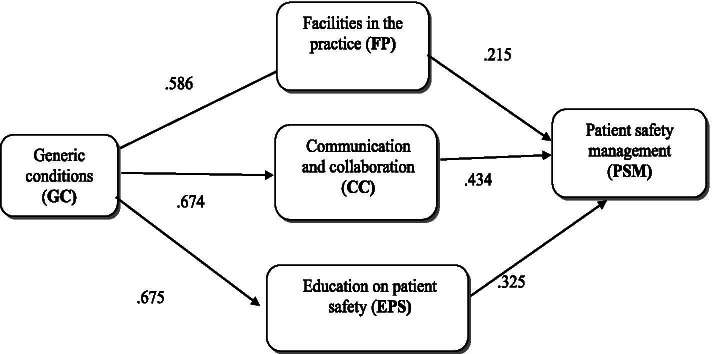
Empirical model. Note: for all *p* < 0.0001

As measures such as effect sizes could not be used in the model, it was decided to determine the strengths of impacts based on standardized path coefficients. Considering the relationships between patient safety management and its three antecedents, the results of our model calculations (Fig. 2) revealed that communication and collaboration had a large effect on patient safety (0.434), facilities in the practice had a small effect (0.215), and education on patient safety had a medium effect (0.325). Consequences as generic conditions for patient safety is large effects of education on patient safety (beta coefficient 0.675), of communication and collaboration (0.674) and of facilities in the practice (0.586). The standardized indirect impact of generic conditions for patient safety on the patient safety management is 0.638. The strength is comparable to the impact of education on patient safety.

### The effect of control variable

The next stage examined whether the significance and strength of this impact was identical in the groups selected in the sample, due to the influence of the sex, age, current professional discipline as GP (general practitioner) and work in counseling centers.

The effect of the facilities in the practice on patient safety management varied between all the groups (Table A2, Supplementary Information); however, the results indicate that facilities in the practice do not have a statistically significant impact on patient safety management in the case of female respondents, those younger than mean age, those who are not GPs and those who did not work in counselling centres. In addition, the results indicate that education on patient safety does improve patient safety management among respondents aged higher than the mean and among those not working in counseling centers.

## Discussion

The aim of this study was to identify the antecedents of patient safety management. Seven dimensions of communication and collaboration, five dimensions of generic conditions for patient safety and eight dimensions of education on patient safety were adapted from previous research [4, 5]. However, only four dimensions were used for practice facilities rather than nine, and seven for patient safety management instead of nine. Each of these dimensions was found to be significant, and were important components of the latent construct. Our findings validated hypotheses H1 and H2 and confirmed that the antecedents of patient safety in primary care are facilities in the practice, communication and collaboration, education and antecedents of them are generic conditions. While all had a positive impact on patient safety, communication and collaboration had the strongest effect, and hence appears to be an important aspect of patient safety management [10].

Primary care modeling of patient safety is complex because causes and effects are interrelated and effects are often mitigated by a set of attributes that cannot be easily modeled using standard techniques. Traditional statistical techniques such as linear regression mainly model the direct impact of uncorrelated variables. Indirect effects are difficult to model and the presence of correlations between explanatory variables may lead to biased coefficient estimates and incorrect model interpretation [19, 26]. Difficulties in measuring patient safety and the complexity of primary care make it desirable to use new methods in the patient safety model [13, 22].

Hypothesis 3 “The relationships with strategies in all subgroups studied are identical” was rejected because only in the case of strategies: general condition and communication and collaboration relationships are identical for each control variables. The impact is different for education and practice facilities strategies.

The literature review recommends choosing a complementary approach through the use of an effective system of reporting incidents from patients [9, 10, 13]. This is not in our model. The most important and pressing action is to implement an information infrastructure that enables capture of adverse events and harm across patient safety systems, thus allowing information to flow freely between GP (general practitioner) and patients on various data platforms [11]. Improved patient safety in primary care results in better quality of care, prevention of injury or harm, and greater patient satisfaction [8].

It has been noted in the literature [1, 9] that reporting incidents, ensuring proper communication among health care providers and fostering a safety culture within the organizational structure allowed medication errors and safety incidents to be minimized, thus improving quality of care in primary healthcare.

Our model shows that it is possible to prevent incidents related to patient safety through professional development and training. The key to improving patient satisfaction is proper communication with the patient and cooperation in the provision of healthcare. Facilities in the practice are also important [8].

## Conclusion

The integrated safety model described in the study can provide a greater insight into patient safety mechanisms. Both our present findings and the literature suggest that one of the leading interventions for improving patient safety management is generic condition empowerment. Involvement can be achieved through facilities, communication and collaboration and education. In all contexts, implementing and sustaining these changes requires management of patient safety focused on collective improvement and teamwork. Our proposed model can achieve this at all levels of the healthcare system. Due to the high importance of patient safety, there are pragmatic reasons for using this model.

### Limitations

The response rate for this study was acceptable, but selection bias cannot be ruled out. Due to the selection procedure used (through a contact person), it is likely that our study included the most experienced patient safety primary care physicians and patient safety experts in the country. Most of the respondents were actually practicing GPs (general practitioners—56.9%), which can be seen as a potential bias. Previous studies [5] indicate that ‘regular’ practicing GPs found patient safety highly relevant, yet they had a very broad idea about patient safety. This survey, based on the results of previous studies, could be used to develop a tool to measure patient safety.

The disadvantage of the study is that SEM should really be used with a large sample and the present study only includes 248 respondents with a very long list of items (38 items, five constructs); this may lead to a model that is over specified. However, the number of samples in the study is by no means exhaustive, and the range of samples could be further expanded to test the accuracy and stability of the results through SEM technology. The use of SEM with latent variables and multiple indicators can produce highly variable estimates in small samples. In the future, it is worth checking the use of a fixed-reliability single indicator models which is recommended in small samples.

In addition, the study was cross-sectional in nature and only one method was used: the perception of the respondents for all study variables. However, it is not known how these strategies actually support the patient safety implementation process.

The cross-sectional nature of the study provides only plausible cause and effect pathways and further research, including prospective studies are needed prior to offering operational advise.

### Practical applications

The proposed model allows antecedents to be identified for locally-rooted planning and implementation of patient safety strategies in primary care and improve understanding of patient safety. Traditionally, these strategies are treated separately; however, this approach has significant disadvantages. Acceptance of the generic conditions for patient safety should generate in this respect: education, facilities, communication and collaboration. This model provides more information on the relationship between patient safety management with practice, communication and collaboration, and contextual factors. Knowledge of antecedents was will help to implement patient safety management, which still needs to improve.

## Supplementary Information

**Additional file 1: Table A1.** Measurement of constructs and characteristic.

**Additional file 2: Table A2.** Standardised values of the estimated parameters in the structural model.

## Data Availability

The dataset supporting the conclusions of this article is available in the Mendeley repository [unique persistent identifier and hyperlink to dataset] https://data.mendeley.com/datasets/bcr9p237g7/draft?a=ff6d6642-ada8-467d-a167-cec151d96e3f.

## Abbreviations

AGFI: Adjusted goodness of fit index
AMOS: Analysis of Moment Structures
AVE: Average variance extracted
CC: Communication and collaboration
CFA: Confirmatory factor analysis
CR: Composite reliability
EPS: Education on patient safety
FP: Facilities in the practice
GC: Generic conditions
GFI: Goodness of fit index
HI: Upper boundary of a two-sided 90% confidence interval for the population RMSEA.
LO: Lower boundary of a two-sided 90% confidence interval for the population RMSEA
PSM: Patient safety management
RMR: Root mean residual
RMSEA: Root mean square of approximation error
SEM: Structural equation modeling
SPSS 20: Statistical Product and Service Solutions 20
χ2/ss: Relative chi-square, $x^{2}$ to degrees of freedom ratios
