# Supplementary material for: Structural equation modeling for identification of patient safety antecedents in primary care
Source: BMC Fam Pract. 2021 Sep 13;22:183. doi: 10.1186/s12875-021-01533-6 (PMC8439075; doi:10.1186/s12875-021-01533-6)
Supplement: Supplementary file 1 — Additional file 1: Table A1. Measurement of constructs and characteristic. [file 12875_2021_1533_MOESM1_ESM.docx]

Supplementary information

Table A1.  Measurement of constructs and characteristic

| Item | Mean | standard deviation | Loading (CFA) | characteristic |
| --- | --- | --- | --- | --- |
| **Facilities in the practice (FP)** | | | | |
| Computerised medical record system which is adequately kept | 2.26 | .968 | - | Cronbach’s alpha=.733  CR=.843  AVE=.576  explain 57.602% of the total variance |
| Reminders and alerts regarding safety issues which are integrated in the medical record system | 1.77 | .980 | .780 |  |
| Computerised decision support regarding medication safety in daily practice | 1.43 | .797 | .797 |  |
| Computerised decision support regarding test ordering in daily practice | 1.34 | .696 | .810 |  |
| Access to web-based clinical guidance tools in daily practice | 1.76 | 1.025 | .635 |  |
| Telephone facilities that allow quick access to the practice particularly for urgent health problems | 1.97 | 1.147 | - |  |
| Planned checks of safety of equipment medication and other facilities in the practice | 2.31 | 1.280 | - |  |
| Working agreements with pharmacists when problems arise with delivering medication e.g. alerts interaction | 1.71 | 1.086 | - |  |
| Forms for reporting incidents available | 1.92 | 1.166 | - |  |
| **Patient safety management (PSM)** | | | | |
| Practice-based reporting and analysis of incidents (e.g. significant event audit) | 1.53 | .908 | - | Cronbach’s alpha =.831  CR=.889  AVE=.503  explain 46.902% of the total variance |
| Reporting and analysis of incidents in small educational groups (e.g. quality circles) | 1.33 | .723 | .782 |  |
| Nationwide or regional educational reporting system for incidents | 1.20 | .530 | .702 |  |
| Nationwide or regional incident reporting weeks | 1.22 | .586 | .716 |  |
| Measurement and feedback on safety culture in general practices | 1.25 | .630 | .657 |  |
| Hygiene protocols and guidelines present | 1.24 | .609 | .699 |  |
| Measurement and feedback on indicators for patient safety | 1.28 | .680 | .645 |  |
| Surveys and other types of consultations of patients regarding safety incidents | 1.32 | .692 | .655 |  |
| Campaigns to increase patients' and public awareness of patient safety in general practice | 1.24 | .588 | .802 |  |
| Periodic audits by an external inspection authority | 1.52 | .922 | - |  |
| **Communication and collaboration (CC)** | | | | |
| Standards for record keeping (ICPC coding electronic records) | 2.21 | 1.082 | .642 | Cronbach’s alpha =.719  CR=.863  AVE=.514  explain 45.08% of the total variance |
| Patient-held medical records | 3.33 | 1.067 | - |  |
| Integrated medical records for communication with specialists and others | 1.43 | .755 | .801 |  |
| Electronic prescriptions and integrated medication overview in the records from the pharmacist | 1.39 | .664 | .706 |  |
| Structured formats for information on referral of patients | 1.81 | 1.083 | .628 |  |
| Comprehensive analysis of prescribing decisions in the pharmacy. using decision support systems | 1.27 | .626 | .745 |  |
| Periodic review of medication by pharmacists in patients who use dangerous (combinations of) medication | 1.32 | .743 | .766 |  |
| **Generic conditions for patient safety (GC)** | | | | |
| Culture and mentality which facilitates learning from incidents | 1.91 | 1.190 | .637 | Cronbach’s alpha=.703  CR=.843  AVE=.520  explain 49.15% of the total variance |
| Understanding of patient safety in health professionals. Particularly regarding how it differs from complications of treatment | 2.15 | 1.245 | .728 |  |
| Workload is perceived as acceptable in general practice | 1.56 | 1.036 | .703 |  |
| Adequate procedures for identifying and managing burn-out in health professionals | 1.25 | .711 | .750 |  |
| Availability of information technology in general practice and skills to use these adequately | 2.15 | 1.108 | .775 |  |
| **Education on patient safety (EPS)** | | | | |
| Education on patient safety in the medical curriculum. before graduation | 1.39 | .787 | .733 | Cronbach’s alpha=.883  CR=.907  AVE=.554  explain 55.44% of the total variance |
| Education on patient safety in the vocational training of GPs (general practitioners) | 1.60 | 1.013 | .749 |  |
| Practical skills workshops for safe practice | 1.22 | .637 | .540 |  |
| Postgraduate education on patient safety of GPs (general practitioners) | 1.36 | .798 | .753 |  |
| Education on patient safety in the nurses curriculum | 1.48 | .935 | .854 |  |
| Education on patient safety in the vocational training of practice nurses | 1.47 | .926 | .861 |  |
| Postgraduate education on patient safety of practice nurses | 1.41 | .853 | .770 |  |
| A guideline on patient safety is available | 1.45 | .876 | .645 |  |

Note: -means the question does not create latent variables (strategies)
