# Supplementary material for: Structural equation modeling for identification of patient safety antecedents in primary care
Source: BMC Fam Pract. 2021 Sep 13;22:183. doi: 10.1186/s12875-021-01533-6 (PMC8439075; doi:10.1186/s12875-021-01533-6)
Supplement: Supplementary file 2 — Additional file 2: Table A2. Standardised values of the estimated parameters in the structural model. [file 12875_2021_1533_MOESM2_ESM.docx]

Supplementary Information

Table A2. Standardised values of the estimated parameters in the structural model

| Sex | Female (*n* = 107) | | Male (*n* = 132) |
| --- | --- | --- | --- |
| FP🡨GC | .627*** | | .519*** |
| EPS🡨GC | .583*** | | .761*** |
| CC🡨GC | .818*** | | .542*** |
| PSM🡨CC | .573** | | .265* |
| **PSM🡨FP** | **.103** | | **.270*** |
| PSM🡨EPS | .258* | | .448*** |
| Current professional discipline GP (general practitioner) | | Yes (*n* = 141) | No (*n* = 107) |
| FP🡨GC | | .557*** | .594*** |
| EPS🡨GC | | .669*** | .690*** |
| CC🡨GC | | .607*** | .736*** |
| PSM🡨CC | | .438** | .464* |
| **PSM🡨FP** | | **.229*** | **.170** |
| PSM🡨EPS | | .295* | .361* |
| Work in counseling centers | | Yes (*n* = 164) | No (*n* = 84) |
| FP🡨GC | | .576*** | .626** |
| EPS🡨GC | | .666*** | .686** |
| CC🡨GC | | .616*** | .757*** |
| PSM🡨CC | | .226* | .746*** |
| **PSM🡨FP** | | **.343**** | **-.006** |
| **PSM🡨EPS** | | **.448***** | **.127** |
| Age | | Lower than mean (*n* = 148) | Higher then mean(*n* = 100) |
| FP🡨GC | | .506*** | .664** |
| EPS🡨GC | | .595*** | .855** |
| CC🡨GC | | .636*** | .710*** |
| PSM🡨CC | | .358** | .649*** |
| **PSM🡨FP** | | **.107** | **.560**** |
| **PSM🡨EPS** | | **.458***** | **-.164** |

*** represent dependencies significant for *p* <0.001, ** *p* <0.01; * *p* <0.05. List of abbreviations: CC: communication and collaboration, EPS: education on patient safety, FP: facilities in the practice, GC: generic conditions, PSM: patient safety management
